# Investigating the Relationship Between COVID-19 and Celiac Disease. A Dual Research Approach

**DOI:** 10.1097/PG9.0000000000000340

**Published:** 2023-08-16

**Authors:** Claudio Tiberti, Margherita Bonamico, Raffaella Nenna, Laura Petrarca, Chiara Maria Trovato, Nicoletta Pietropaoli, Valeria Fassino, Fabio Midulla, Andrea Lenzi, Salvatore Oliva, Monica Montuori

**Affiliations:** From the * Department of Experimental Medicine; † “Sapienza” Foundation; ‡ Department of Maternal, Infantile and Urological Sciences; § Department of Translational and Precision Medicine, “Sapienza” University of Rome, Italy; ∥ Hepatology Gastroenterology and Nutrition Unit, Bambino Gesù Children Hospital, Rome, Italy.

**Keywords:** anti-transglutaminase antibodies, celiac disease, CD, COVID-19, SARS-CoV-2antibodies

## Abstract

**Background:**

Most evidence on the coronavirus disease 2019 (COVID-19) pandemic, has been obtained from web- or telephone-based surveys. In particular, few laboratory data, often incomplete, have been reported on the frequency of COVID-19-related serology at celiac disease (CD) diagnosis or on the effects of COVID-19 on the development of CD-specific autoimmunity.

**Objectives:**

The objective of this retrospective cross-sectional case/control study was to: (1) evaluate the frequency of severe acute respiratory syndrome coronavirus 2 (SARS-CoV-2) antibodies in 78 children and adolescents at CD diagnosis (CD, 44 females, median age 7.4 years); (2) evaluate the frequency of IgA-anti-transglutaminase antibodies (IgA-tTGAbs) in 97 nonceliac patients (50 females, median age 9.0 years) who contracted SARS-CoV-2 infection during the pandemic (February–April 2021). As a control (CTRL) group, we analyzed 141 healthy subjects (79 females, median age 9.8 years) enrolled during the pandemic.

**Methods:**

SARS-CoV-2 IgM- and IgG-antibodies were detected by chemiluminescent microparticle immunoassays. IgA-tTGAbs were detected by a fluid-phase radioimmunoassay.

**Results:**

Six out of 78 (7.7%) CD patients tested positive for SARS-CoV-2Abs, with a frequency not significantly different from CTRL subjects (9.2%). None of the 97 nonceliac COVID-19 patients tested positive for IgA-tTG antibodies.

**Conclusion:**

These 2 distinct research approaches showed (1) similar frequencies of SARS-CoV-2 immunoreactivities in CD patients and CTRL subjects and, (2) no ability of SARS-CoV-2 to induce a CD-specific immune response, at least in the 3–4 months following SARS-CoV-2 infection.

**What Is Known**

-  Several evidence already present in the literature suggest that viral infections may play a role in promoting celiac disease (CD).
-  Although coronavirus disease 2019 (COVID-19) is mainly defined by its respiratory symptoms, it can also affect the digestive system, causing
- gastrointestinal symptoms such as diarrhea, nausea, vomiting, and abdominal pain.
-  Most of the studies that have examined the relationship between COVID-19 and CD have relied on web- or telephone-based surveys for data collection.
-  To date, only a few, incomplete, and conflicting laboratory data have been reported on COVID-19-related serology at CD diagnosis.

**What Is New**

-  During the pandemic, newly diagnosed CD patients tested positive for severe acute respiratory syndrome coronavirus 2 antibodies with a frequency not significantly different from that of healthy control subjects.
-  COVID-19 patients did not develop celiac-specific autoimmunity, at least in the first months after severe acute respiratory syndrome coronavirus 2 infection.

## INTRODUCTION

Celiac disease (CD) is an immune-mediated gluten-sensitive enteropathy elicited by the ingestion of gluten in genetically predisposed individuals (1). Although gluten is unquestionably the driver for CD, indirect evidence suggests that viruses may play an essential role in the pathogenesis of CD (2,3). Coronavirus disease 2019 (COVID-19), caused by severe acute respiratory syndrome coronavirus 2 (SARS-CoV-2) and responsible for the ongoing pandemic, is predominantly a respiratory disease (4). However, COVID-19 can also affect the digestive system, causing gastrointestinal symptoms such as diarrhea, loss of appetite, nausea, vomiting, and abdominal pain. Several mechanisms have been proposed to explain the gastrointestinal tract involvement in COVID-19 (5,6). Among these, the ability of SARS-CoV-2 to enter enterocytes via the angiotensin-converting enzyme-2 receptor leads to a direct cytopathic effect, increasing intestinal barrier permeability and the passage of gliadin in the intestinal lamina propria (7). The COVID-19 pandemic and subsequent infection control measures imposed by governments have had a significant impact on CD clinical practice and pediatric gastroenterology practice, not only on patients affected by SARS-CoV-2 infection. The potential consequences of reduced health care access due to fear of attending the hospitals during the COVID-19 pandemic include delayed diagnosis and a more severe illness presentation, as reported in 2 cases of CD presented with celiac crisis (5). To date, despite the potential relevance for CD clinical practice, the impact of SARS-CoV-2 on CD incidence is not clear (8–10). Although COVID-19’s impact has been investigated in other autoimmune diseases (11), only web- or telephone-based survey data have been reported on the frequency of COVID-19 immunity in CD patients or on the development of CD autoimmunity following COVID-19 infection. The study of the humoral immune response against SARS-CoV-2 can provide a measure of the exposure of CD patients to the virus, whereas the detection of celiac autoimmunity in COVID-19 patient sera may be a valid approach to understanding whether the viral infection may eventually induce CD autoimmunity. Based on these considerations, the aims of our study were:

1. To evaluate the frequency of SARS-CoV-2 IgM- and IgG-antibodies in newly diagnosed CD patients during the pandemic and compare the relative results to those found in a population of healthy subjects recruited from the hospital outpatient clinic (control [CTRL]).

2. To evaluate the frequency of IgA-anti-transglutaminase antibodies (IgA-tTGAbs) in a cohort of non-CD children and adolescents who contracted SARS-CoV-2 infection during the pandemic.

## METHODS

The serum samples utilized in the study were collected from a total of 318 individuals and subdivided as follows (see also Table 1):

**TABLE 1.** Demographic characteristics of the three groups of patients

	Celiac disease	Controls	COVID-19
Number	78	141	97
Median age (years)	7.4	9.8	9.0
Age range (years)	1.7–16.4	1.1–17.9	0.4–17.0
Females/males	44/34	79/62	50/47

COVID-19 = Coronavirus disease 2019.

1. We enrolled 78 newly diagnosed CD patients, including 44 females and 34 males with a median age of 7.4 years (range 1.7–16.4 years), who were on a gluten-containing diet. Of the 78 patients, 35 (44.9%) had a biopsy-confirmed CD diagnosis showing 3B and 3C duodenal mucosa lesions according to the modified Marsh-Oberhuber classification (12). The remaining 43 patients (55.1%) were diagnosed based on the more recent ESPGHAN 2012 guidelines, with tTG-IgA levels ≥10 times the upper limit of normal (13), and the 2020 Guidelines without HLA typing (14). Among the newly diagnosed CD patients, 49/78 (62.8%) had a typical disease presentation (ie, gastrointestinal symptoms such as chronic diarrhea, weight loss, constipation, and abdominal pain), while 20/78 (25.6%) had atypical presentation of the disease (characterized by extraintestinal manifestations such as iron deficiency, osteoporosis, and short stature). Seven out of 78 patients (9.0%) were asymptomatic, and their CD was detected through family screening. The CD patients were consecutively enrolled from November 2019 to December 2021 at the Department of Maternal, Infantile, and Urological Sciences, Policlinico Umberto I, “Sapienza” University of Rome, Italy.

2. A total of 141 healthy subjects were sequentially recruited from the hospital outpatient clinic from November 2019 to December 2021 (CTRL). The CTRL group consisted of 79 females and 62 males with a median age of 9.8 years (age range 1.1–17.9 years). At the time of recruitment, all CTRL subjects tested negative for SARS-CoV-2 through an oropharyngeal swab test. The CTRL subjects, as well as CD patients, were consecutively enrolled from November 2019 to December 2021 at the Department of Maternal, Infantile, and Urological Sciences, Policlinico Umberto I, “Sapienza” University of Rome, Rome, Italy. These 141 healthy subjects were the control group compared to the newly diagnosed CD patients for the frequency of SARS-CoV-2Abs. The same healthy subjects, except those who tested positive for SARS-CoV-2Abs, were the group of controls compared with COVID-19 patients for the presence of IgA-tTGAbs.

3. In total 99 COVID-19 children and adolescents contracted the disease during the second wave of the pandemic. All these patients underwent an oropharyngeal swab to receive a diagnosis of SARS-CoV-2 infection. Two celiac patients on a gluten-free diet were excluded from the study and the remaining 97 patients (50 females and 47 males) had a median age of 9.0 years (range 0.4–17.0 years). During COVID-19, 82.5% of patients were symptomatic, and 94.8% tested positive for SARS-CoV-2Abs. The mean interval between the first SARS-CoV-2 positive oropharyngeal swab and the medical examination was 114 days (range 34–332 days). Serum samples for the detection of IgA-tTG antibodies were collected between February and April 2021.

No one of the subjects enrolled in the study had been vaccinated against SARS-CoV-2 before blood sample collection. No individuals with IgA deficiency were included in the study. The study received approval from the local Ethics Committee (reference number 0399/2021). For this cross-sectional case/control study, the sera of CD, CTRL, and COVID-19 cases were analyzed for the presence of SARS-CoV-2 IgM/IgG and IgA-tTG antibodies.

### SARS-CoV-2 IgM and IgG Detection

To detect IgM- and IgG-antibodies to SARS-CoV-2, we utilized the chemiluminescent microparticle SARS-CoV-2 IgM (code SR87, Abbott) and IgG (code SR86, Abbott) immunoassays on the ARCHITECT i System (Abbott). Both assays were designed to detect antibodies to the nucleocapsid protein of SARS-CoV-2. The chemiluminescent reaction produced a relative light unit (RLU), which directly correlated with the amount of IgM- or IgG-antibodies to SARS-CoV-2 in the serum sample. To determine the presence or absence of IgM- or IgG-antibodies, we compared the RLU in the reaction (S) to the RLU of the relative calibrator (C). Serum samples were considered SARS-CoV-2 IgM- and IgG-antibody-positive if the Ab-indexes (S/C) were ≥1.0 and ≥1.4, respectively.

### IgA-tTGAb Detection

We detected serum IgA-tTGAb using a fluid-phase radioimmunoprecipitation method. This involved transcribing and translating the full-length human tTG cDNA in vitro in the presence of 35-S methionine (15). This assay was reported to be the most sensitive and specific assay for CD in the First International Transglutaminase Autoantibody Workshop (16). Autoantibody levels were expressed as an Ab-index, which was calculated as follows: (sample cpm − negative standard sample cpm)/(positive standard sample cpm − negative standard sample cpm). We considered serum samples tTGAb positive if the Ab-index was above 0.025.

## STATISTICAL ANALYSIS

We expressed data as medians or means ± standard deviation for continuous variables and as proportions for categorical variables. To compare groups we used Student’s t-test or Fisher exact test. A two-tailed P-value <0.05 was considered statistically significant. All statistical analyses were performed using SPSS 21.0 for Windows.

## RESULTS

### IgM- and IgG-SARS-COV-2 antibodies in CD and CTRL subjects

Table 2 presents the results of our study on IgM- and IgG-SARS-CoV-2 antibodies. We found SARS-CoV-2Abs in 6/78 (7.7%) of newly diagnosed CD patients and in 13/141(9.2%) of CTRLs (*P* = ns). CD patients tested positive only for SARS-CoV-2 IgG-antibodies, whereas 5.7% of CTRL subjects tested positive for SARS-CoV-2 IgG and 4.3% for SARS-CoV-2 IgM antibodies. Of the 13 CTRL subjects who tested positive for SARS-CoV-2Abs, only 1 had both IgG and IgM SARS-CoV-2 antibodies. SARS-CoV-2 antibodies were equally distributed among CD females and males (50%), as well as among CTRL subjects (53.8%).

**TABLE 2.** SARS-CoV-2 IgG and IgM in celiac disease patients and healthy control subjects

	Celiac disease patients (n = 78; 44 female, 34 male)				Healthy control subjects* (n = 141; 79 female, 62 male)			
	IgG+	IgG−	IgM+	IgM−	IgG+	IgG−	IgM+	IgM−
n° Ab+	6	72	0	78	8	133	6	135
%	(7.7)	(92.3)	(0)	(100)	(5.7)	(94.3)	(4.3)	(95.7)
Mean age	7.6 ± 5.6	8.1 ± 4.2	-	8.1 ± 4.7	9.3 ± 3.7	9.7 ± 4.8	9.7 ± 4.8	9.5 ± 4.8
Female/male	3/3	41/31	-	44/34	4/4	75/58	3/3	76/59
Mean Ab titer	3.1 ± 0.1	0.1 ± 0.1	-	0.1 ± 0.01	4.3 ± 2.3	0.1 ± 0.2	3.0 ± 2.5	0.2 ± 0.1

SARS-CoV-2 = severe acute respiratory syndrome coronavirus 2.

*1 healthy control subject was positive for both IgG and IgM SARS-CoV-2Abs.

Table 3 shows that newly diagnosed CD patients who tested positive for SARS-COV-2 antibodies had slightly lower IgA-anti-transglutaminase antibody titers (0.314 ± 0.23) than negative CD patients (0.560 ± 0.35) (*P* = ns). Table 4 presents the characteristics of the 6 CD patients who tested positive for SARS-CoV-2 antibodies at CD diagnosis. Only 1 of the 6 CD patients had a typical presentation (16.7%). Interestingly, 3 out of the 6 patients who tested positive for SARS-CoV-2 antibodies showed diabetes or diabetes-related features (1 had type 1 diabetes since 2014, another was positive for type 1 diabetes-specific autoantibodies, and the third had hyperglycemia).

**TABLE 3.** tTGAb in celiac disease patients, COVID-19 patients, and healthy control subjects

	Celiac disease patients (n = 78; 44 female, 34 male)		COVID-19 patients (n = 97; 50 female, 47 male)		Healthy control subjects (n = 141; 79 female, 62 male)	
	SARS-CoV-2 Ab+	SARS-CoV-2 Ab−	SARS-CoV-2 Ab+	SARS-CoV-2 Ab−	SARS-CoV-2 Ab+	SARS-CoV-2 Ab−
n° tTGAb+	6/6	72/72	0/91	0/6	0/13	0/128
(%)	(100)	(100)	(0)	(0)	(0)	(0)
Ab titer (mean)	0.314 ± 0.23	0.560 ± 0.35	−0.033 ± 0.02	−0.030 ± 0.03	−0.066 ± 0.05	−0.026 ± 0.03

COVID-19 = Coronavirus disease 2019; SARS-CoV-2 = severe acute respiratory syndrome coronavirus 2.

**TABLE 4.** Characteristics of the 6 newly diagnosed celiac disease patients positive for SARS-CoV-2Abs

	Sex	Age (years)	CD clinical presentation	Marsh-Oberhuber score	tTG Abs	SARS-CoV-2Abs		COVID-19 symptoms	Other features
						IgM	IgG		
1	Female	8.1	Atypical	3C	+	−	+	Yes	
2	Female	4.1	Atypical	3B	+	−	+	Yes	T1DM Ab+
3	Male	14.2	Asymptomatic	3C	+	−	+	Yes	T1DM
4	Female	1.7	Typical	No biopsy*	+	−	+	Yes	
5	Male	14.5	Atypical	3B/3C	+	−	+	Yes	
6	Male	3.1	Asymptomatic	3B	+	−	+	Yes	Hyperglycemia

CD = celiac disease; COVID-19 = Coronavirus disease 2019; SARS-CoV-2 = severe acute respiratory syndrome coronavirus 2.

*Diagnosed according to ESPGHAN 2020 guidelines (ref 14).

### IgA-tTGAbs in COVID-19 Patients

None of the 97 non-celiac patients who contracted COVID-19 during the pandemic tested positive for IgA-tTG antibodies.

### IgA-tTGAbs in SARS-CoV-2 Ab Negative CTRL Patients

Table 2 shows that out of the 141 CTRL subjects enrolled, 128 tested negative for SARS-CoV-2 antibodies and served as the CTRL group of noninfected individuals. All 128 of these patients also tested negative for IgA-tTG antibodies.

### IgA-tTGAbs in SARS-CoV-2Ab Positive CTRL Patients

Table 2 shows that out of 141 CTRL subjects studied, 13 tested positive for SARS-CoV-2 antibodies. These individuals may have contracted COVID-19 in the months before our study, in addition to the 97 COVID-19 patients who were investigated. It is worth noting that none of the SARS-CoV-2 antibody-positive control subjects tested positive for IgA-tTG antibodies.

## DISCUSSION

This study reports immunological data on the relationship between COVID-19 and CD, using 2 distinct research approaches and including cohorts of patients/controls not yet vaccinated against SARS-CoV-2. Most scientific publications that evaluate the impact of COVID-19 disease on CD have focused on data collected through interviews or population-based analysis and have reported clinical, biochemical, and histological findings (8–10,17–20). However, to the best of our knowledge, only a few, incomplete, and conflicting laboratory data have been published on this topic. We found that studies investigating the frequency of SARS-CoV-2 humoral immune response in CD patients have only focused on adults with long-term CD (17–19). However, no information is available on this topic for CD children. Additionally, it is unclear whether COVID-19 may trigger CD autoimmunity in nonceliac patients. This study provides the first evidence in these fields. We found that 7.7% of patients with CD at the time of diagnosis had tested positive for SARS-CoV-2 antibodies. However, this percentage was not significantly different from that found in healthy controls without CD who were tested during the same pandemic period. To date, only 2 studies (18,19) have investigated the presence of SARS-CoV-2 antibodies in CD patients. However, despite both studies enrolling cohorts of adult CD patients on a gluten-free diet during similar periods of the 2020 pandemic (February–June and April–June), the results were significantly different. One study found SARS-CoV-2 antibodies in only 2.8% of the cases (18), while the other study found them in 18% of cases (19). These different results may be due to differences in the design and execution of the two studies. However, we believe that the reduced number of sera compared with the number of patients recruited may also explain these discrepancies. Schiepatti et al (18) analyzed only 24 sera out of the 324 recruited patients (7.4%), while Elli et al (19) analyzed 109 sera (30.1%) of the 362 patients who were contacted by phone and agreed to be tested for SARS-CoV-2 antibodies after lockdown. It is not possible to compare the results of these studies to the findings in our study (7.7%), as our study involved newly diagnosed CD children and adolescents who were not on a gluten-free diet. Additionally, the study was conducted over a longer pandemic period (November 2019–December 2021), and the patients had not been selected. Interestingly, we found 6 CD patients who tested positive for SARS-CoV-2 antibodies, and of those 6 patients, 3 had features that could be attributed to diabetes. One patient has had type 1 diabetes since 2014, another was positive for one type 1 diabetes-specific autoantibody, and the last patient had hyperglycemia at the time of CD diagnosis. This finding is consistent with previous studies that have suggested a possible link between COVID-19 and the onset of type 1 diabetes (21,22). Additionally, Cakir et al (23) reported an increased association of CD with type 1 diabetes during the pandemic. However, due to the relatively small number of CD patients, we investigated, further studies with a larger sample size will be required to confirm any potential relationship between COVID-19, CD, and type 1 diabetes.

The second aim of our study was to assess the prevalence of CD-specific IgA-tTG antibodies in a cohort of children and adolescents who contracted COVID-19 during the pandemic. The detection of CD autoimmunity in COVID-19 patients is a valid approach to understand whether the viral infection may act as a trigger for CD. However, we found that all 97 COVID-19 patients and all 128 healthy subjects investigated were negative for IgA-tTG antibodies, which suggests that there is no association between COVID-19 and CD. CD is a disease that usually affects 1–2% of the general population (24). Our finding that all 13 healthy control subjects who tested positive for SARS-CoV-2 antibodies were negative for IgA-tTG antibodies supports these data. It should be noted that our results are based on patients who were analyzed shortly after being diagnosed with COVID-19, within 3–4 months.

In conclusion, our study, which used 2 different research approaches to investigate the relationship between COVID-19 and CD, yielded the same result: there appears to be no link between COVID-19 and the onset of CD. This conclusion is supported by the finding that our cohorts of newly diagnosed CD patients and healthy subjects were affected by the COVID-19 disease in similar proportions during the pandemic and that patients with COVID-19 did not develop CD autoimmunity within the first months following virus infection. However, longer-term follow-up studies of SARS-CoV-2 infected patients will be necessary to fully exclude any association between COVID-19 and the development of CD.

## ACKNOWLEDGMENTS

The authors would like to express their gratitude to Lisa Magdalene Cole for editing and feedback of this article.
